# Case Report: Venous crossover bypass to the contralateral internal mammary vein to restore venous outflow in revision surgery in autologous free flap breast reconstruction

**DOI:** 10.3389/fsurg.2026.1932861

**Published:** 2026-09-25

**Authors:** Wibke Mueller-Seubert, Isabell Scherrer, Raymund E. Horch, Andreas Arkudas

**Affiliations:** Department of Plastic and Hand Surgery, University Hospital Erlangen, Friedrich Alexander University Erlangen-Nuernberg FAU, Erlangen, Germany

**Keywords:** autologous free flap breast reconstruction, cephalic vein, crossover bypass, flap revision, internal mammary vein

## Abstract

Breast reconstruction using free flaps is a standardized procedure in specialized centers. Despite a very low flap failure rate, total flap loss has devastating consequences for affected patients. Venous congestion remains one of the most common causes of flap compromise, requiring revision surgery. Herein, we present a salvage technique for revision surgery to restore venous outflow using a crossover bypass to the contralateral internal mammary vein (IMV). Our patient had undergone multiple previous oncological surgeries, including a partial sternum resection. As the ipsilateral IMV proved insufficient, the ipsilateral cephalic vein was initially used as a recipient vein. Because of subsequent venous congestion, the cephalic vein was repurposed as a crossover bypass to the contralateral IMV. Following this procedure, the flap healed without further complications. A venous crossover bypass to the contralateral internal mammary vessels represents a reliable salvage option, even in complex revision surgery.

## Introduction

Autologous breast reconstruction using abdominal free flaps [deep inferior epigastric perforator- or muscle-sparing transverse rectus abdominis (msTRAM)-flap] is a safe and standardized procedure with a very low flap failure rate of less than 5% (1–6). Nevertheless, flap loss in autologous breast reconstruction is a devastating consequence with an emotionally difficult experience for women (7). The main microsurgical complication resulting in flap loss is venous congestion or arterial compromise (1), and therefore, an adequate revision strategy is of paramount importance. In the case of venous congestion, thrombectomy and additional venous anastomosis of the superficial inferior epigastric vein are the most common strategies with a successful revision rate up to 60% (2, 4, 8). Arterial revision includes thrombectomy as well with a similar successful revision rate described between 20% and 60% (2, 4). If the local vessels are not suitable for reanastomosis, other solutions are required (9). Herein, we present the case of a patient with successful flap revision after venous congestion with a cephalic vein crossover bypass to the contralateral internal mammary vein.

## Case history

A 54-year-old patient (Figure 1) presented to our hospital after recurrent breast cancer of the left breast. As first-line treatment, the patient received breast-conserving therapy with adjuvant chemotherapy for breast cancer on the left side. This was followed by a sternum resection with subsequent sternum stabilization using a Gore-Tex mesh plate 1 year later. After recurrence, mastectomy was performed by removing a parasternal left chest wall metastasis, with adjuvant radiochemotherapy performed another 5 years later. In addition, a port catheter had been placed previously in the left subclavian vein for chemotherapy. Breast reconstruction using autologous tissue was planned nearly 2 years after the last operation. The patient had a body mass index of 31,6 kg/m^2^ and no history of smoking or diabetes.

**Figure 1:**
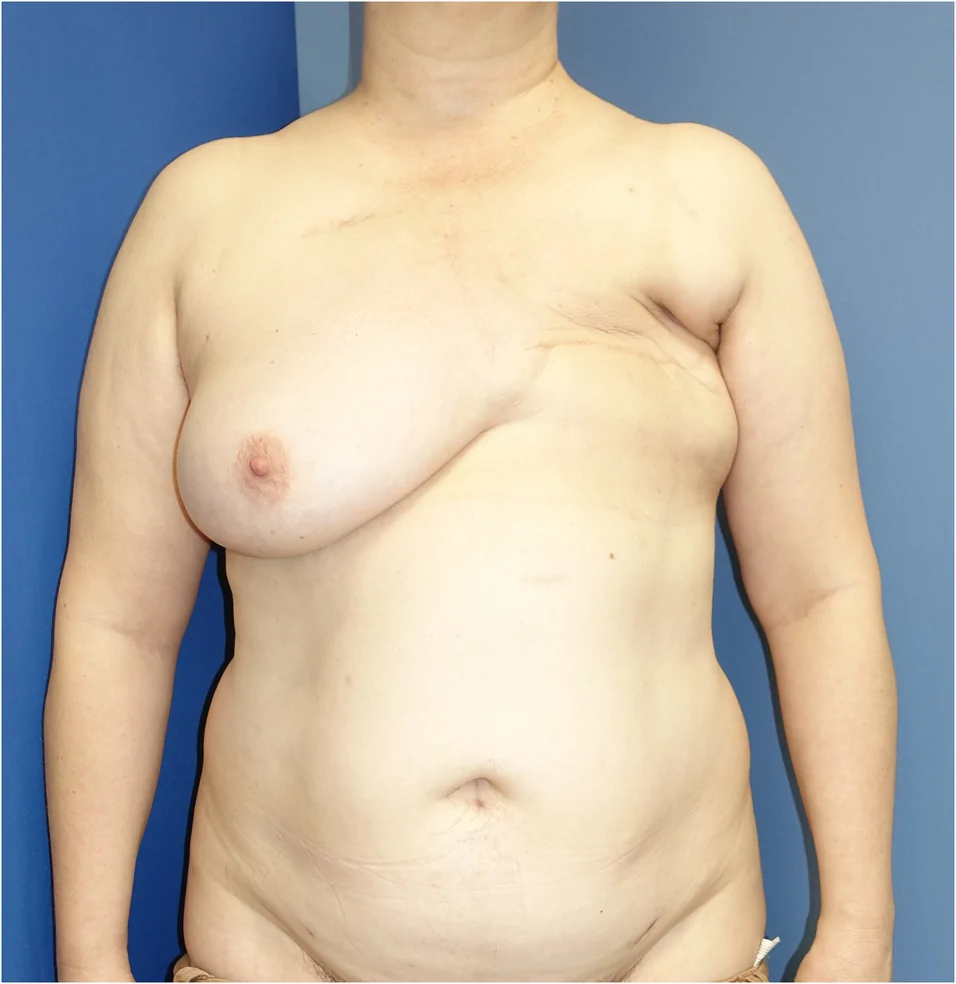
54 years old patient after breast cancer on the left side preoperatively.

## Methods

Because of the multiple previous operations, a CT angiography (CTA) of the local recipient vessels was performed, which showed that both the internal mammary artery (IMA) and the thoracodorsal artery were properly contrasted. Furthermore, we performed a CT angiography of the abdomen to visualize the perforators. The abdomen was chosen as the donor site because of tissue excess.

In accordance with the procedure of CT angiography, an ms2-TRAM flap was harvested from the left abdomen including one thin medial and lateral perforator each 3 cm below the umbilicus. During the process of preparing the internal mammary vessels after removing the fourth rib, as part of our standardized approach to prevent the formation of a dent by removing a more cranial rib, the IMV especially presented itself as very thin and fragile. After flap harvest, an arterial anastomosis was performed using Ethilon 8-0 end-to-end to the IMA. Because of the small size of the recipient IMV, a venous anastomosis of the flap´s deep venous system was performed using a 2.0-mm vein coupler after intraoperative measurement (Synovis Micro Companies Alliance, Birmingham, AL, USA). The flap ischemia time was 83 min. After a short time, venous compromise was observed. Intraoperative indocyanine green angiography with the microscope-integrated indocyanine green-based video angiography (Kinevo 900, Carl Zeiss, Oberkochen, Germany) also revealed insufficient venous drainage via the IMV. In order to achieve sufficient venous drainage, the cephalic vein from the ipsilateral arm was harvested by a vertical incision from the deltopectoral groove to the elbow. A length of 15 cm of the vessel was dissected, tunneled subcutaneously to reach the flap as the recipient vein. Subsequently, a venous anastomosis was performed using a 3.0-mm coupler. The port catheter was removed from the subclavian vein (Figure 2).

**Figure 2:**
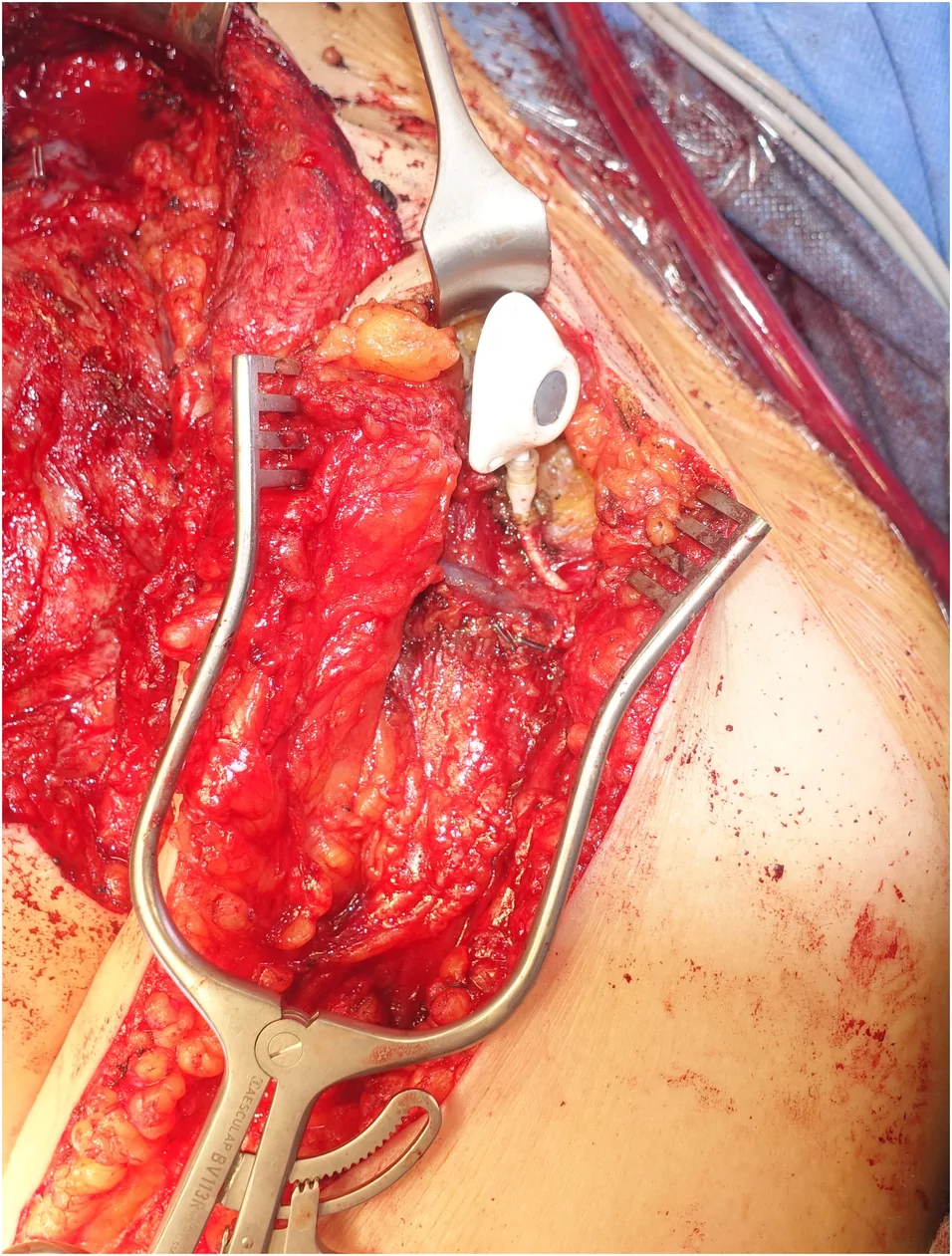
Removal of port catheter from the left subclavian vein after venous anastomosis to the left cephalic vein.

A clinical examination and indocyanine green angiography now showed adequate venous outflow. A total of 6 h after the operation, flap revision became necessary because of venous congestion. Multiple thrombi were found in the cephalic vein, which were removed using a Fogarty catheter (size 2F; 0.67 mm). After a skin incision on the contralateral side, the fourth rib was partially removed and the contralateral mammary vessels were exposed and presented with a sufficient size. A subcutaneous presternal tunnel was created and an end-to-end anastomosis was performed between the contralateral IMV, the cephalic vein as crossover bypass, and the flap vein using a 3.0-mm coupler for each vein (Figure 3).

**Figure 3:**
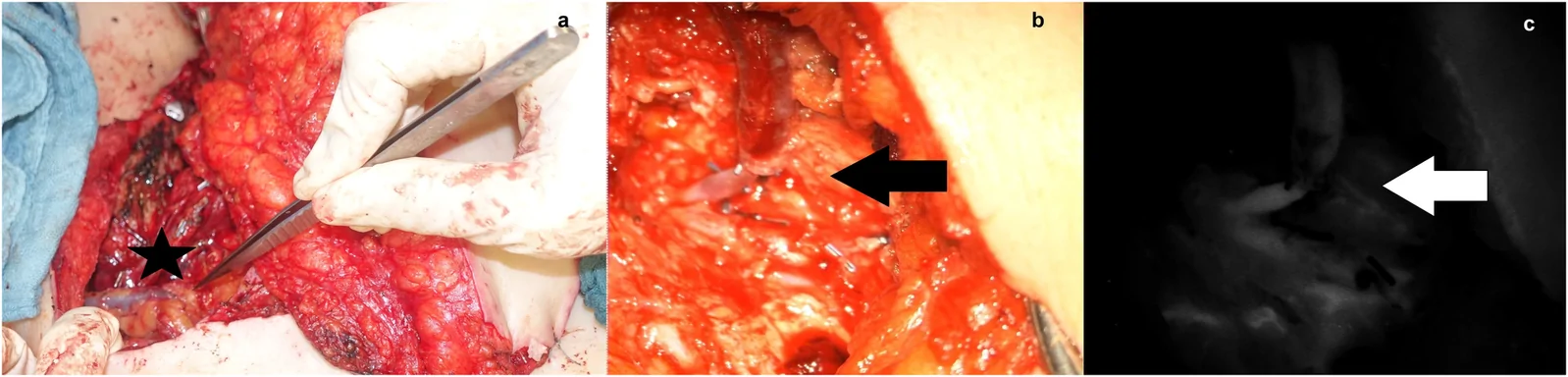
Venous crossover bypass (marked with *) between the flap (a) and the contralateral IMV (arrow pointing to coupler anastomosis, b), indocyanine green angiography showed adequate venous outflow (arrow pointing to coupler between cephalica vein and contralateral IMV, c).

A clinical examination and indocyanine green angiography revealed adequate venous outflow. The patient received postoperative anticoagulation with intravenous unfractionated heparine (activated partial thromboplastin time (aPTT) 45 s) and was continued with Phenprocoumon (INR 2–2.5) for 6 months postoperatively.

The duration of the first operation was 599 min and that of the second operation was 272 min.

## Result

The patient was discharged 11 days after revision surgery; no further complications were noticed 6 months postoperatively (Figure 4). A symmetrical breast reduction of the contralateral side is now planned.

**Figure 4:**
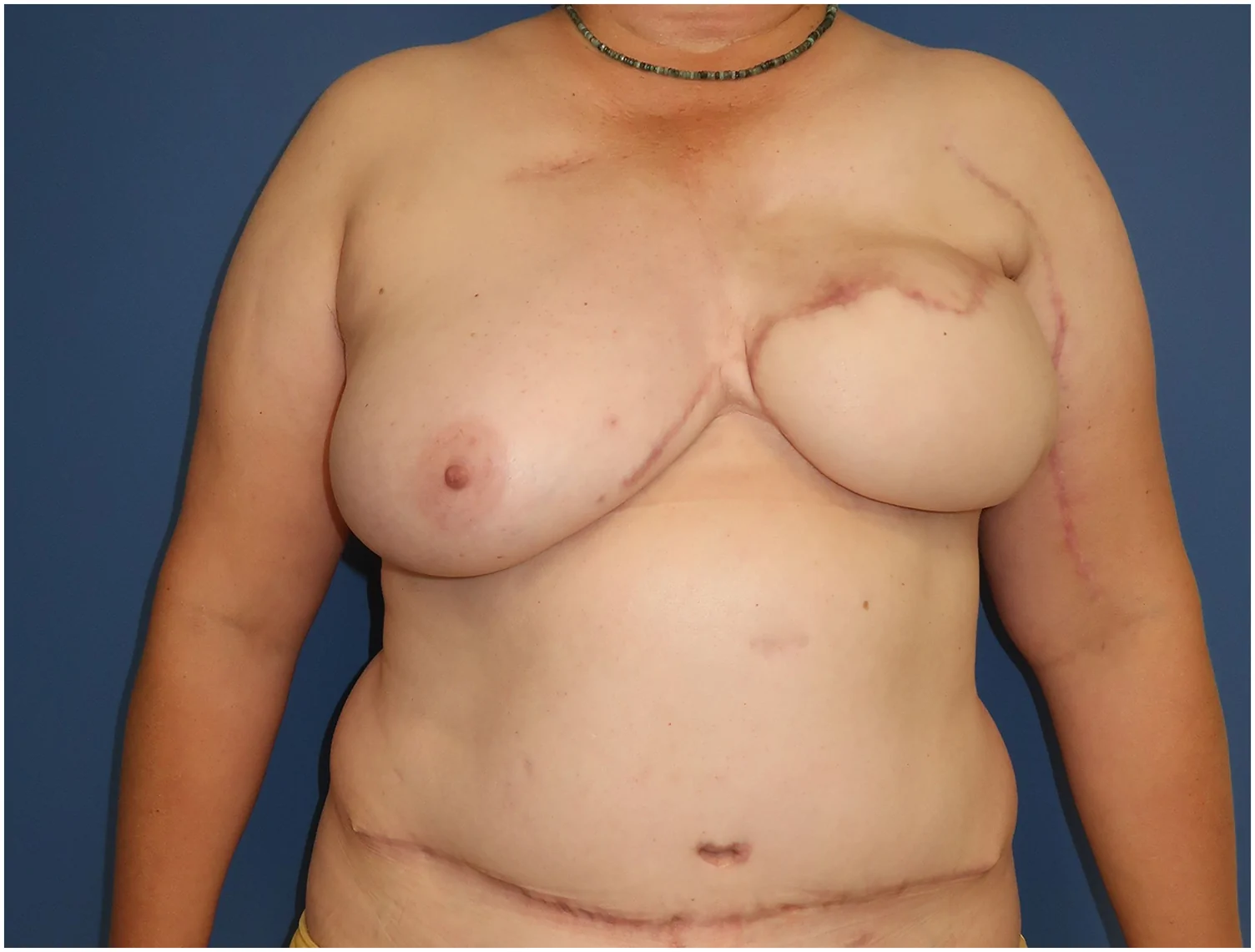
6 months postoperatively after successful revision surgery, a symmetrical breast reduction of the contralateral side is planned.

## Discussion

Breast reconstruction with autologous tissue is a standardized procedure in specialized centers. The ms2TRAM-flap was chosen in this patient because of tissue excess in the abdomen. Other gluteal or lumbar perforator flaps or stacked deep inferior epigastric perforator flaps can be chosen if there is a lack of sufficient abdominal tissue (10–12).

Nonetheless, in rare cases of arterial congestion or venous compromise, prompt surgical revision is essential to prevent flap loss. Venous congestion may often be resolved through revision of the anastomosis, thrombectomy, or additional venous outflow via the superficial inferior epigastric vein (2, 4, 13). In the present patient case, these conventional strategies were not feasible due to insufficient local recipient veins. Although dissection to a more cranial intercostal space may provide a larger IMV or a venous confluence suitable for anastomosis (14, 15), extensive prior irradiation and scarring rendered the ipsilateral IMV unsuitable, even at higher rib levels. Moreover, a reliable preoperative assessment of IMV quality using CTA remains challenging.

The cephalic vein has been established as a reliable recipient vein for additional venous drainage in breast reconstruction (14, 16, 17). Despite the presence of a previously placed port catheter in the left subclavian vein, venous drainage proved initially adequate after a cephalic vein anastomosis, as confirmed clinically and by indocyanine green angiography. Thrombotic occlusion occurred within the cephalic vein itself rather than the subclavian vein, and the port catheter was removed to minimize potential obstruction.

Although both the internal mammary and thoracodorsal vessels are considered safe recipient options (18), the internal mammary vessels are often preferred because of their consistent anatomy, larger caliber, favorable flap medialization, lower radiation–induced fibrosis, and higher negative venous pressure (19, 20). In this patient, the thoracodorsal system was intentionally preserved as a potential salvage option using a pedicled latissimus dorsi flap (21).

Additional venous outflow through the perfused bone marrow has been described in a case report to increase the chances of venous return and reduce the risk of venous stasis for a defect at the distal upper extremity (22). This may be a salvage option in non-irradiated areas with sufficient, healthy bone nearby. But this maybe a safe alternative for use in autologous breast reconstruction—especially in patients who have previously undergone radiation therapy and may have a sclerosis of the sternum. Use of the contralateral internal mammary vessels as rescue recipient vessels has been previously reported in selected cases in which ipsilateral vessels were inadequate (23). Previously described techniques include venous crossover bypass using the saphenous vein (24, 25) or direct pedicle transposition through a sternal groove without interposition grafting (23). However, these reports describe only single-stage salvage procedures (24, 25). Theoretically but without sufficient data to prove the claim, a higher risk of venous collapse may be entailed when the vein or the venous graft is placed between the skin and the sternum to the contralateral side. A bony groove in the anterior sternal wall may reduce this risk. On the other hand, scooping a bony groove may increase postoperative pain and surgical time.

To the best of our knowledge, this is the first report describing the use of the cephalic vein as a crossover bypass to the contralateral internal mammary vein in unilateral breast reconstruction during revision surgery.

## Conclusion

This case demonstrates that venous anastomosis to the contralateral internal mammary vein using a venous crossover bypass is a reliable salvage option, even in complex free flap revision surgery. The contralateral internal mammary vessels should be considered a valuable rescue option in unilateral autologous breast reconstruction.

## Data Availability

The raw data supporting the conclusions of this article will be made available by the authors, without undue reservation.
